# How Attachment to Dogs and to Other Humans Relate to Mental Health

**DOI:** 10.3390/ani14192773

**Published:** 2024-09-26

**Authors:** Katherine Northrope, Matthew B. Ruby, Tiffani J. Howell

**Affiliations:** 1 School of Psychology and Public Health, La Trobe University, Bendigo 3552, Australia; t.howell@latrobe.edu.au; 2 School of Psychology and Public Health, La Trobe University, Melbourne 3086, Australia; m.ruby@latrobe.edu.au

**Keywords:** dog-owner bond, mental illness, pet-owner relationships, social support

## Abstract

**Simple Summary:**

This study explored how people’s relationships with their dogs were associated with mental health and how this compared to their relationships with other humans. Our results found that owners who had a strong attachment to their dogs tended to have poorer mental health outcomes. The connection between a strong attachment to dogs and poor mental health may be partly due to these owners having an anxious attachment style towards other people, suggesting these owners may turn to their dogs for emotional support due to a lack of dependable human connections. The relationship between a strong attachment to dogs and poorer mental health was also influenced by gender, with this relationship being significant for women, but not for men. Together, these results highlight a potential risk to mental health for owners who rely on their strong bond with their dogs to compensate for feeling anxious about their relationships with other humans. For these owners, it may be worth considering how they can improve their connections with other humans to be able to better support their mental health.

**Abstract:**

It is unclear how pet ownership is related to mental health, with some previous research suggesting pet owners have better mental health, while other research suggests they have worse mental health. Some researchers have suggested that it may be more useful to investigate the bond people feel with their pets and how this may impact mental health; however, this too has led to mixed results. This study examined how owners’ attachment to their dogs was associated with mental health and how this compared to their attachment relationships with other humans in a sample of 607 dog owners. Our findings indicate that both strong and insecure attachments to dogs are linked to poorer mental health outcomes, as was having an insecure attachment style in their human relationships. The adverse impact of strong attachment to dogs on mental health was mediated by these owners having an anxious attachment style toward other people, which in turn was associated with poorer mental health. The relationship between a strong attachment to dogs and poorer mental health was also moderated by gender, with this relationship being significant in women but not significant for men. Together, these results suggest a possible risk to mental health for owners who form a strong attachment to their dogs to compensate for anxious attachments in human relationships.

## 1. Introduction

There is some research evidence to suggest that pet owners have better physical and mental health [1,2,3], but the overall evidence does not consistently show these benefits [4]. Other research indicates that pet owners have higher rates of anxiety and depression and poorer physical health than non-owners [5,6,7]. One explanation for these mixed findings is that much of the research focuses only on whether people have pets rather than considering the relationship they have with the pet [8,9,10,11]. Indeed, one study found that new dog owners reported better general health than did non-owners, and this effect was stronger for those who were more attached to their dog [12]. This is consistent with attachment theory, where secure attachment styles in relationships with other people are associated with better mental health outcomes [13].

Attachment theory was proposed by Bowlby [14] to explain the instinctual bond that human infants form with their mothers. While this attachment system is most evident in infancy and early childhood for primary caregivers, it is active in other relationships across the lifespan, for example with friends and romantic partners [15]. In adulthood, those who are higher in attachment-related avoidance have a tendency for emotional distance in relationships, whereas those who are higher in attachment-related anxiety desire closeness and may become distressed if they think their partner is not readily available [15]. Those who are low on both attachment anxiety and avoidance are classified as being secure in their attachment style, as they feel comfortable being close to other people and do not worry about being abandoned by others. Those who have a secure attachment style tend to have better well-being [16]. This is partly due to their positive attitudes about their ability to cope and their positive attitudes about other people being available to support them when they need it [17].

There is also evidence to suggest that pets may be seen as an attachment figure [18]. The relationship between attachment to pets and well-being, however, is also mixed [19]. While some studies find that a stronger attachment to pets is associated with better mental health [20], other research finds that it is associated with worse mental health [10,21]. One explanation of this negative relationship is that a stronger attachment to one’s pet may be a compensatory mechanism for low levels of social support [22,23], which in turn leads to poorer mental health outcomes [24]. One German study found that the relationship between higher attachment to one’s pet and poorer mental health outcomes was fully mediated by participants’ insecure attachment to humans [24]. This study used the Lexington Attachment to Pets Scale (LAPS) [25] to measure the owners’ attachment to their dogs. While this scale measures the strength of the bond and is the most commonly used measure of attachment to pets [26], it has been critiqued as not fully capturing attachment as described in attachment theory [8].

The Pet Attachment Questionnaire (PAQ) is a self-report scale based on traditional attachment theory orientations designed to assess attachment orientations in the human-pet relationship and measures two factors of anxiety and avoidance [18]. Pet attachment anxiety was found to be negatively associated with psychological well-being and positively associated with psychological distress even after controlling for participants’ attachment styles to other humans [18]. Lass-Hennemann et al. [24] note that future studies should explore whether it is the strength of the attachment towards the pet (i.e., how the LAPS measures attachment) or the style (i.e., how the PAQ measures attachment) that is most important when considering the mental health of pet owners.

The majority of participants in Lass-Hennemann et al. [24] were (92.79%) women. Women tend to report higher attachment to their pets compared to men, as measured by the LAPS [25,27,28,29]. In contrast, no gender differences were found for the PAQ for attachment avoidance or anxiety [18]. Gender differences in adult attachment towards humans have not been consistently shown. Some studies find that men and women do not differ in their levels of avoidant or anxious attachment [30,31,32,33], although a meta-analysis found that men had higher levels of avoidant attachment and women had higher levels of anxious attachment, although these effects were small and varied across samples [34]. Men and women also have similar rates of experiencing mental illness [35], although differences exist for particular disorders. Women are more likely to experience anxiety and depression [36,37], whereas men experience higher rates of drug and alcohol abuse [38,39] and are at a higher risk of suicide [40]. These gender differences bring into question whether the results of Lass-Hennemann et al.’s [24] study would apply to men.

This study is a replication of Lass-Hennemann et al.’s [24] study in English with a gender-balanced sample, with the addition of the Pet Attachment Questionnaire (PAQ) [18]. In Lass-Hennemann et al. [24], human attachment is measured by the Revised Attachment Adult Scale (R-AAS; [31]), which measures closeness and dependence, which are seen as an (inverse) measure of avoidant attachment, and anxiety, which is a measure of anxious attachment style. The aim of this study is to test whether there is a relationship between attachment to pet dogs and mental health and if this is mediated by human attachment in both men and women. We pre-registered the study’s materials, aims, and hypotheses with the Open Science Framework (available online: https://osf.io/4vdhb, accessed on 27 May 2024) prior to data collection.

**Hypothesis** **1a.** *Dog owners with stronger attachments to their dogs would have poorer mental health outcomes*.

**Hypothesis** **1b.** *Dog owners who had insecure attachments in human relationships would have poorer mental health outcomes*.

**Hypothesis** **1c.** *Dog owners who had stronger attachments to their dogs would have poorer mental health outcomes, but this would be mediated by their attachments to other humans*.

**Hypothesis** **2.** *Dog owners with insecure attachments to their dogs would have worse mental health outcomes than those with secure attachments to their dogs*.

**Hypothesis** **3.** *Men would score lower on attachment to their dogs compared to women*.

To explore gender effects, we compared men and women on the attachment towards their dogs as measured by the LAPS and PAQ, adult attachment style, and mental health outcomes. Given the mixed results for gender regarding adult attachment style and mental health, we did not hypothesize the direction of any differences for these outcomes. We also did not expect any gender differences for attachment style towards pets as measured by the PAQ.

## 2. Materials and Methods

### 2.1. Participants

In total, 607 dog owners completed the survey. Participants were recruited online via Prolific (Prolific Academic Ltd., London, UK) by targeting those who had previously indicated that they owned a dog, were aged 18 or older, and spoke English. Participants were geographically diverse, including 123 (20.3%) from South Africa, 85 (14%) from Mexico, 68 (11.2%) from the United States, 64 (10.5%) from Canada, 54 (8.9%) from Australia, 44 (7.2%) from Chile, and 33 (5.4%) from the United Kingdom, with the remaining 136 (22.4%) from 29 different countries. All participants could read and write in English. Other participant information is presented in Table 1.

### 2.2. Materials

The survey materials used in this study are the same scales used by Lass-Hennemann et al. [24], with the addition of the PAQ. After completing the Participant Information and Consent Form, participants completed the following measures in the order below:

### 2.3. Demographics and Information about Dog Ownership

Participants provided demographic information, including gender, age, education, country of residency, marital status, and whether any other adults or children live in their household. They also answered questions about their pet dog. If they owned more than one dog, they were instructed to respond about their favorite dog. Items included the dog’s age and size, and how much time they spent interacting with their dog in a day. Participants reported the presence of any other pets living in the house.

### 2.4. Pet Attachment Questionnaire

The Pet Attachment Questionnaire (PAQ) [18] consists of 26 items that ask owners about the security of their attachment with their pet and has been used for both cat and dog owners. Participants rate their level of agreement with the 26 statements about their relationship with their dog and rate the extent to which each item describes their feelings and thoughts in this relationship on a 7-point scale ranging from 1 (not at all) to 7 (very much). Two subscales correspond with attachment anxiety and avoidance, which consist of 13 items each. An example item from the anxiety factors is “I feel frustrated if my pet doesn’t seem to be available for me when I need it”, and an example item from the avoidance factor is “I prefer not to be too close to my pet”. Negatively phrased items are reverse scored such that higher scores for each subscale indicate higher levels of attachment anxiety and avoidance, respectively (possible subscale range: 13–91). Internal consistency was high for both scales in our study (anxiety α = 0.86, avoidance α = 0.87).

### 2.5. Lexington Attachment to Pets Scale

The Lexington Attachment to Pets Scale (LAPS) [25] consists of 23 items that ask owners about their bond with their pet and has been used for both cat and dog owners. Participants rate their level of agreement with the 23 statements about their favorite pet on a 4-point scale from 0 (strongly disagree) to 3 (strongly agree). An example item is “my pet and I have a very close relationship”. Scores for the 23 items are added together to create a total score after reversing any negatively worded items. Higher scores indicate a stronger attachment to the pet (possible range: 0–69). Internal consistency for the 23 items in our study was high (α = 0.92).

### 2.6. The Brief Symptom Inventory

The Brief Symptom Inventory (BSI) [41] is a 53-item self-report measure that assesses symptomatic distress using nine subscales. Participants indicate how much they had been bothered by the list of symptoms over the previous 7 days on a 4-point Likert scale, ranging from 0 (not at all) to 4 (extremely). A Global Severity Index (BSI GSI) is the sum of these items. This measure is suitable for non-clinical samples. Higher scores indicate more severe symptomology (possible range: 0–212). Internal reliability was excellent in our study (α = 0.98).

### 2.7. The Revised Adult Attachment Scale

The Revised Adult Attachment Scale (R-AAS) [42] is an 18-item scale that measures individual differences in attachment style through three subscales, each with six items. The closeness subscale measures how comfortable a person is with closeness; an example item is “I find it relatively easy to get close to others”. The dependence subscale measures the extent to which a person feels comfortable depending on others; an example item is “I find it difficult to allow myself to depend on others”. The anxiety subscale measures how anxious a person is about being rejected or abandoned by others; an example item is “I often worry that romantic partners don’t really love me”. Participants respond on a 5-point scale, from 1 (not at all characteristic of me) to 5 (very characteristic of me). Negatively worded items were reversed so that higher scores indicate higher levels of closeness, dependence, and anxiety, respectively (possible subscale range: 6–30). Internal reliability for these were acceptable to excellent in our study (closeness α = 0.74, dependence α = 0.78, anxiety α = 0.90).

### 2.8. Procedure

The study was approved by the Human Research Ethics Committee of La Trobe University (HEC24113). We recruited participants through Prolific (Prolific Academic Ltd.) in May 2024. On average, it took participants 14 min to complete the survey. Questionpro was programmed so that it was not possible for responses to be incomplete. They were paid GBP 1.80 in accordance with Prolific’s payment guidelines.

### 2.9. Data Analysis

Data were analyzed using IBM SPSS Statistics v29 (IBM, Armonk, NY, USA). Subscale and total scores were calculated for each of the scales after reversing the appropriate items. Data were first screened for outliers that were 3 SD above or below the mean. Twenty-eight participants were identified as outliers for their responses on either the PAQ avoidance, LAPS, or BSI GSI. Analyses were conducted with and without these outliers. Including these outliers had little impact on results, so we decided to report all analyses with these included. Linearity was confirmed using scatterplots. Histograms were then examined for the LAPS, PAQ subscales, R-AAS subscales, and the BSI GSI. The LAPS, BSI GSI, and PAQ avoidance were non-normally distributed.

Correlation analyses were run to test if poorer mental health outcomes were associated with stronger attachments to dogs (Hypothesis 1a), lower scores on closeness and dependence, and higher on anxiety in human relationships (Hypothesis 1b), and with insecure attachments to dogs (Hypothesis 2). The correlation analysis was completed with PAQ subscales, R-AAS subscales, LAPS overall score, and the BSI Global Severity Index (GSI). Due to some of the variables being non-normally distributed, the non-parametric Spearman’s Rho correlations are reported.

To test Hypothesis 1c that dog owners who had stronger attachments to their dogs would have poorer mental health outcomes, but that this would be mediated by their attachments to other humans, a mediation analysis was conducted to test whether attachment to one’s pet (i.e., LAPS) is related to BSI GSI scores and whether this is mediated by the R-AAS subscales of closeness, dependence, and anxiety scores. This was conducted using PROCESS custom dialog box v4.2 [43]. For regression analyses, including mediation and moderation, violations of normality are unlikely to affect the validity of statistical inferences unless the sample size is very small [43]. The effects were estimated based on 5000 bootstrap samples.

To test Hypothesis 3 that men would score lower on attachment to their dogs compared to women (as measured by the LAPS), a *t*-test was conducted. Due to the small number of non-binary participants, they were excluded from this analysis. Possible gender differences for PAQ anxiety and avoidance, R-AAS closeness, dependence, and anxiety and BSI GSI scores were also examined. *t*-tests are robust to data that are not normally distributed in samples of 30+ participants [44].

These first three hypotheses and their analyses were pre-registered on the Open Science Framework (OSF). Any additional analyses should be considered exploratory. An exploratory analysis was conducted to determine if the relationship between the LAPS and BSI GSI was moderated by PAQ anxiety and avoidance. Another exploratory analysis was conducted to determine if the relationship between the LAPS and BSI GSI was moderated by gender for men and women. As above, non-binary participants were excluded from this analysis comparing men and women. These were also done using PROCESS. The significance level for all analyses in this study is *p* < 0.05 (two-tailed). Where the assumption of the homogeneity of variance was violated as indicated by Levene’s test for equality of variances, the results were taken from the “Equal variances not assumed” section on SPSS. All effects for the mediation and moderation analyses are reported as unstandardized, as recommended by Hayes [43]. It should be noted that our mediation model does not imply that attachment to dogs causally affects attachment to humans but is used to explore how these factors may be related to mental health. Similarly, we are unable to infer causality from our moderation models but have used these to explore relationships between the variables.

## 3. Results

Information about the dog is presented in Table 2. On average, participants left their dog alone for 4.48 h a day (SD 3.61) and interacted with their dogs (e.g., playing, walking, cuddling on the sofa) for 5.24 h (SD 5.24).

A correlation analysis was completed with PAQ subscales, R-AAS subscales, LAPS Total Score, and the BSI Global Severity Index (GSI). These results are presented in Table 3. Scores on R-AAS closeness and dependence were negatively associated with scores on BSI GSI, with medium effect sizes [45]. Scores on the LAPS and PAQ avoidance were positively associated with BSI GSI, with small effect sizes. Scores on PAQ anxiety and R-AAS anxiety were also positively associated with BSI GSI with a medium and large effect size, respectively.

*t*-tests results to compare men and women on all variables are reported in Table 4. Women scored significantly higher than men on the LAPS, R-AAS anxiety and BSI GSI at the *p* < 0.05 level. Men scored significantly higher in PAQ avoidance. These were all small effects [45]. Due to the large number of comparisons, we applied a Bonferroni correction, dividing the normal significance level of *p* < 0.05 by the number of variables, which reduced the significance level to *p* < 0.007. In this case, the gender difference for R-AAS anxiety would no longer be considered significant.

### 3.1. Mediation

**Figure 1:**
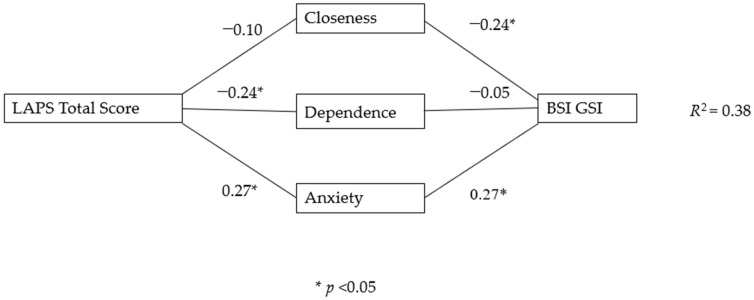
Model of LAPS on BSI GSI as mediated by R-AAS subscales.

To determine if the relationship between the LAPS and the BSI GSI was mediated by adult attachment style, a model was tested with the R-AAS subscales of closeness, dependence, and anxiety subscales as mediators. Higher scores on the LAPS were associated with higher R-AAS anxiety and lower scores on R-AAS dependence, but were not significantly associated with R-AAS closeness. The total effect of the LAPS on BSI GSI was significant, *b* = 0.20, 95% CI [0.07, 0.33], *p* = 0.002. Including the R-AAS subscales reduced the direct effect of the LAPS on BSI GSI to be non-significant (*b* = 0.09, 95% CI [−0.01, 0.19], *p* = 0.077). The indirect effects were estimated based on 5000 bootstrap samples using a percentile confidence interval method. We found a significant total indirect effect that mediated the relationship between the LAPS and BSI GSI through R-AAS anxiety, *b* = 0.07, 95% CI [0.03, 0.13]. Higher scores on the LAPS were associated with higher R-AAS Anxiety, which in turn was associated with higher scores on the BSI GSI. There was no significant indirect effect through R-AAS closeness *b* = 0.02, 95% CI [−0.01, 0.06], or through R-AAS dependence *b* = 0.01, 95% CI [0.00, 0.03]. The pattern of relationships in this model was consistent with the correlations reported in Table 3. However, the relationship between R-AAS dependence and BSI GSI was no longer significant when controlling for the other R-AAS variables. This mediation model is presented in Figure 1. The *R*^2^ for this model was 0.38, explaining 38% of the variance in levels of BSI GSI. This is in line with the R^2^ of Lass-Hennemann et al. [24] of 0.36.

### 3.2. Moderation

As an exploratory analysis, we also investigated whether the relationship between the LAPS and BSI GSI was moderated by attachment style towards the dog using PAQ anxiety and avoidance scores. These variables were first mean centered. PAQ avoidance (see Table 5) and anxiety (see Table 6) did not significantly moderate the relationship between the LAPS and BSI GSI. The *R*^2^ for the PAQ avoidance model was 0.12 and for the PAQ anxiety model was 0.15, explaining 12 and 15% of the variance in BSI GSI scores, respectively.

**Figure 2:**
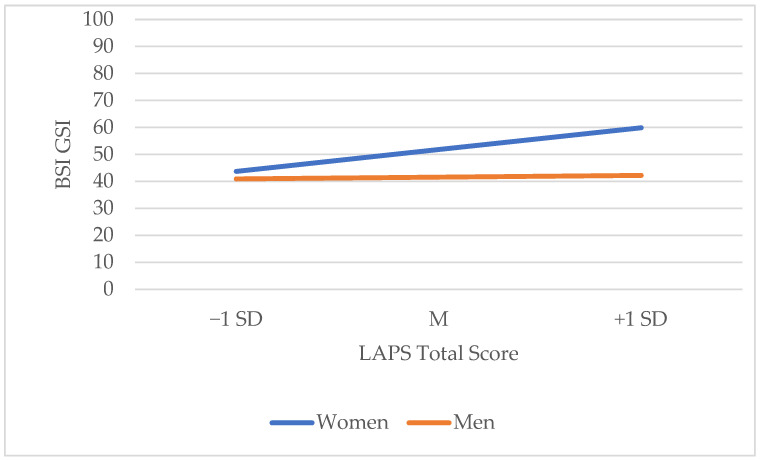
Simple slopes equations of the regression of LAPS on BSI GSI for men and women.

As an exploratory analysis, we also investigated whether the relationship between the LAPS and BSI GSI was moderated by gender. The continuous variables were first mean centered. Gender did significantly moderate the relationship between the LAPS and BSI GSI (see Table 7). For men, there was a non-significant relationship between the LAPS and BSI, *b* = 0.06, 95% CI [−0.34, 0.46], *t* = 0.30, *p* = 0.767. For women, there was a significant positive relationship between the LAPS and BS GSI, *b* = 0.76, 95% CI [0.32, 1.19], *t* = 3.43, *p* ≤ 0.001. Simple slopes are presented, demonstrating this relationship in Figure 2. The *R*^2^ for this model was 0.04, explaining 4% of the variance.

## 4. Discussion

The aim of this study was to test whether there is a relationship between attachment to pet dogs and mental health, and if this is mediated by human attachment, in both men and women. Hypothesis 1a, that dog owners with stronger attachments to their dogs (as measured by the LAPS) would have poorer mental health outcomes, was supported. This is consistent with Lass Hennemann et al. [24], who found owners with stronger attachments to their dogs had worse mental health in their German sample of mostly women. It is also consistent with other research that found that a stronger attachment to pets is associated with worse mental health [21,46,47].

Hypothesis 1b, that dog owners who scored lower on closeness and dependence, and higher on anxiety (as measured by the AAS) would have poorer mental health outcomes, was also supported. Again, this is consistent with results from Lass-Hennemann et al. [24]. This is also in line with research finding that attachment anxiety and avoidance are associated with higher levels of depression and anxiety, although this relationship tends to be stronger for those with anxious attachment styles [48,49].

Hypothesis 1c was that dog owners with stronger attachments to their dogs (as measured by the LAPS) would have poorer mental health outcomes, but this would be mediated by their attachments to other humans (as measured by the AAS). This was partially supported, with R-AAS anxiety mediating the relationship between the LAPS and BSI GSI scores, but no mediation occurred through R-AAS closeness or dependence. This is similar to the results of Lass-Hennemann et al. [24], although they found the relationship between the LAPS and BSI GSI was mediated by both R-AAS anxiety and dependence but not R-AAS closeness. While those who scored lower on R-AAS dependence and closeness did tend to have poorer mental health, they also scored lower on attachment towards their dogs as measured by the LAPS, which means we would not expect these variables to explain the relationship between stronger attachment to dogs and poor mental health.

Dog owners with an anxious attachment style in human relationships may be compensating for their desire to form an attachment with humans, whom they perceive as unreliable, by forming a stronger attachment to their dogs. For those with anxious attachment styles, pets may be seen as a less threatening attachment figure [50]. In contrast, those with an avoidant attachment style in human relationships also avoided forming a strong attachment to their dogs in our study. Those with avoidant attachment styles may be able to cope with day-to-day life stress, but find that they are unable to cope with more extreme forms of stress like divorce or illness where support from other people may be necessary [17]. In comparison, those with anxious attachment styles become distressed when they feel that their attachment figure is not readily available and are more likely to ruminate or behave in self-defeating ways, which further contributes to their risk of poor mental health [13,17]. This suggests that the relationship between having a strong attachment to one’s pet and poorer mental health may be less about the relationship with the pet and more about those with an anxious attachment style feeling like they do not have a reliable human attachment figure to support them during times of stress. For some people, their strong attachment to their dogs may be due to them feeling like their dog is the only reliable social support [51].

Hypothesis 2, that dog owners with insecure attachments to their dogs (as measured by the PAQ) would have worse mental health outcomes than those with secure attachments to their dogs, was supported. This is in line with previous research that found that these insecure attachments towards pets are associated with worse mental health outcomes [18,52]. Including the PAQ extends the work of Lass-Hennemann et al. [24] by answering the question of how degree (as measured by the LAPS) and style (as measured by the PAQ) of attachment to pet dogs relate to mental health. The correlation for PAQ anxious attachment and poorer mental health was twice the size compared to the LAPS score and PAQ avoidant attachment, suggesting that anxiously attached owners are at a greater risk of mental health problems. This is consistent with research showing that anxious attachments in human relationships are associated with an increased risk of mental distress [13,17].

We also compared men and women on the attachment towards their dogs as measured by the LAPS and PAQ, adult attachment style, and mental health outcomes. Hypothesis 3, that men would score lower on attachment to their dogs compared to women as measured by the LAPS, was supported. This is consistent with previous research findings that women are more attached to their pets [25,27,28,29]. Women also scored higher than men on R-AAS anxiety (although this was no longer significant with the Bonferroni correction) but did not differ on R-AAS closeness or dependence. This is supported by evidence that women are more likely to have an anxious attachment style, and that gender differences for avoidance attachment are inconsistent across study samples [34].

Women scored higher than men on BSI GSI, indicating that women in our study had worse mental health outcomes. This is consistent with evidence that women experience higher levels of anxiety and depression [36,37], which are two aspects of mental health that the BSI measures. Unexpectedly, men scored significantly higher in PAQ avoidance, which is inconsistent with Zilcha-Mano et al. [18], which found no gender differences. However, this is consistent with men generally having a lower attachment to the pets and being more likely to have an avoidant attachment style in human relationships [34]. One reason our results may differ from Zilcha-Mano et al. [18] is due to differences in how participants were recruited. Zilcha-Mano et al. [18] recruited pet owners from the community without monetary reward, meaning participants likely took part in the study because they were highly motivated to share their experiences of pet ownership, which may in turn be associated with both men and women being more likely to have a secure attachment towards their pets. Together, these gender comparisons extend the research by Lass-Hennemann et al. [24] to explore how men and women differ on these outcomes and how this may be affecting the relationship between attachment to pet dogs and mental health.

As part of an exploratory analysis, we investigated whether the relationship between the LAPS and BSI GSI was moderated by attachment style towards the dog using PAQ anxiety and avoidance scores. Neither of these significantly moderated this relationship. We did find that gender significantly moderated the relationship between the LAPS and BSI GSI, with stronger attachment to dogs being associated with worse mental health for women, but not for men.

Together, these results provide additional insight into why associations between pet ownership and mental health are inconsistent and why those with a stronger attachment to their pets may be at risk for poorer mental health. Women were more likely to have a strong attachment to their pets and have an anxious attachment style in their human relationships, which may be why the relationship between a strong attachment to their dogs and poorer mental health was significant for women only. Owners who have a strong attachment to their dog may be seeking comfort that they do not feel is available in their human relationships. However, dogs may not be able to provide the emotional support that their owners require, particularly during times of distress where more practical support may be needed. For these owners, it may be worth considering how they can improve their connections with other humans to be able to better support their mental health.

The strengths of this study are that we had a gender-balanced sample, allowing us to explore the effects of attachment to pets and mental health that are more representative of both men and women. We also used the PAQ to investigate attachment to pets, which more closely aligns with attachment theory. As we recruited participants via Prolific, our sample may be more representative of pet owners, as we may have been able to recruit participants who would not normally be interested in taking part in studies about their pets due to having a weaker, more avoidant attachment to their dog. This also allowed us to recruit a geographically diverse sample, with a substantial number of participants living in South Africa and Mexico. At the same time, the geographical diversity of our sample limits any generalizations that can be made to specific geographic populations, as pets may fulfill different roles in different cultures. Other limitations of this study are that it cross-sectional, meaning that we cannot draw any causal inferences in the direction of the relationship between variables. This is particularly relevant for the finding that a stronger attachment to one’s dog is associated with worse mental health, as it is not clear whether this attachment is causing this poorer outcome or whether there are other factors like the owners’ human relationships or other risk factors that are influencing both their relationship with their dog and mental health.

Future research should extend the results of this study to explore owners of pets other than dogs. It would be interesting to explore, for example, if cat owners who have an anxious attachment style in their human relationships also compensate by forming a stronger attachment with their cats, and how this relates to mental health, particularly as there is some evidence to suggest that cat owners have poorer mental health than dog owners [53,54], and are less attached to their cats [25,29]. Other research is also needed to support our exploratory analysis, which found that the relationship between stronger attachment to dogs and poorer mental health was significant for women but not for men. It is unclear at this stage whether this pattern was unique to our sample or is consistent with dog owners more generally, and what factors may be influencing these gender differences. Qualitative research may be helpful to better understand how owners feel about their relationship with their pets and how it relates to their mental health, and how this compares to their human relationships. It would also be useful to explore how the dog, and in particular the dog’s attachment to their owner [55], may be impacting this relationship and contributing to the owner’s mental health. Our dataset is also available for any researchers interested in exploring the relationship between variables in the study further (available online: https://osf.io/69zjp/?view_only=9e3bdcf3fbed4e9dbce8ca3e370d06f7, accessed on 27 May 2024).

## 5. Conclusions

In conclusion, owners who have a stronger attachment (as measured by the LAPS) and who have an insecure attachment (as measured by the PAQ) to their dogs had poorer mental health. This relationship may at least partially be explained by these people having insecure attachments to other humans, particularly those with an anxious attachment style, which is contributing to poorer mental health. This may particularly be of concern for women, who tended to have a stronger attachment to their dogs and more anxious attachment styles, which may place them at a greater risk for mental health problems.

## Figures and Tables

**Table 1:** Demographics for human participants.

Gender	*n*	%
Woman	288	47.4
Man	303	49.9
Non-binary	14	2.3
Other	1	0.2
Prefer not to say	1	0.2
Age	Mean: 32 years 1 month	Range: 18–79 years
**Education**	***n***	**%**
Primary or middle school	6	1
High school	126	20.8
Technical/trade school	67	11
University	396	65.2
Other	9	1.5
Prefer not to say	3	0.5
**Marital Status**	***n***	**%**
Single or never married	307	50.6
Living with a partner but not married	132	21.7
Married	142	23.4
Separated	4	0.7
Divorced	8	1.3
Widowed	3	0.5
Other	6	0.8
Prefer not to say	5	1
**Number of other adults in home**	***n***	**%**
0	49	8.1
1	224	36.9
2	160	26.4
3	98	16.1
4	45	7.4
5+	24	4
Prefer not to say	7	1.2
**Number of children in home**	***n***	**%**
0	338	55.7
1	132	21.7
2	95	15.7
3	27	4.4
4	5	0.8
5+	4	0.7
Prefer not to say	6	1

**Table 2:** Characteristics of favorite dog and other pets.

Dog Sex	*n*	%
Male (entire)	225	37.1
Male (desexed/neutered)	121	19.9
Female (entire)	127	20.9
Female (desexed/spayed)	134	22.1
Dog Age	Median: 5 years	Range: <1–16 years
**Dog Size**	***n***	**%**
Small (less than 30 lbs/14 kg)	242	39.9
Medium (30 lbs/14 kg–55 lbs/25 kg)	267	44
Large (more than 55 lbs/25 kg)	98	16.1
**Other pets in home**	**Yes**	**No**
	327 (53.9%)	280 (46.1%)
**Types of other pets**	***n***	**%**
Cat	201	33.1
Dog	158	26
Bird	39	6.4
Rabbit	22	3.6
Other	52	8.6

**Table 3:** Sperman rho correlations for study variables.

Variable	M (SD)	1	2	3	4	5	6
1. LAPS	53.23 (10.63)	-					
2. PAQ avoidance	21.87 (9.03)	−0.54 *	-				
3. PAQ anxiety	35.71 (12.89)	0.37 *	0.10	-			
4. R-AAS closeness	18.83 (4.89)	−0.08	−0.15 *	−0.18 *	-		
5. R-AAS dependence	15.93 (5.06)	−0.17 *	−0.02	−0.20 *	0.49 *	-	
6. R-AAS anxiety	16.38 (6.71)	0.12 *	0.13 *	0.34 *	0.43 *	−0.51 *	-
7. BSI GSI	47.99 (39.44)	0.13 *	0.19 *	0.39 *	−0.49 *	−0.44 *	0.57 *

Note. * Correlation is significant at the *p* < 0.01 level. Lighter shades of orange indicate small positive correlations (0.10 to 0.29), medium shades of oranges indicate medium positive correlations (0.30 to 0.49), darker shades of orange indicate large positive correlations (≥0.50). Lighter shades of blue indicate small negative correlations, medium shades of blue indicate negative correlations, darker shades of blue indicate large negative correlations.

**Table 4:** Differences by gender for study variables.

	Men	Women					
	M (SD)	M (SD)	Mean Difference	df	*t*	*p*	d
LAPS	51.71 (10.85)	54.76 (10.31)	3.05	589	3.50	<0.001	0.29
PAQ avoidance	22.95 (9.28)	20.66 (8.59)	−2.28	588.60 *	−3.11	<0.001	0.26
PAQ anxiety	35.05 (12.45)	36.40 (13.38)	1.35	589	1.27	0.103	0.10
R-AAS closeness	19.14 (4.92)	18.56 (4.90)	−0.57	589	−1.42	0.078	0.12
R-AAS dependence	15.90 (4.91)	15.95 (5.25)	0.45	589	0.10	0.458	0.01
R-AAS anxiety	15.69 (6.53)	17.02 (6.81)	1.33	589	2.43	0.008	0.20
BSI GSI	41.47 (36.61)	52.96 (40.94)	11.49	589	3.60	<0.001	0.30

Note. * indicates equal variances not assumed.

**Table 5:** Moderation analysis of LAPS × PAQ avoidance on BSI GSI.

	*b*	SE B	*t*	*p*
Constant	1.89	0.03	60.37	<0.001
LAPS	0.63	0.08	7.95	<0.001
PAQ avoidance	0.41	0.08	7.95	<0.001
LAPS × PAQ avoidance	−0.08	0.07	−1.31	0.191

Note. *R*^2^ = 0.12.

**Table 6:** Moderation analysis of LAPS × PAQ anxiety on BSI GSI.

	*b*	SE B	*t*	*p*
Constant	1.89	0.03	63.85	<0.001
LAPS	0.01	0.07	0.09	0.926
PAQ anxiety	0.28	0.03	9.10	<0.001
LAPS × PAQ anxiety	0.08	0.05	1.36	0.176

Note. *R*^2^ = 0.15.

**Table 7:** Moderation analysis of LAPS × gender on BSI GSI.

	*b*	SE B	*t*	*p*
Constant	62.00	5.10	12.16	<0.001
LAPS	1.45	0.49	2.99	0.002
Gender	−10.22	3.20	−3.20	0.002
LAPS × Gender	−0.69	0.30	−2.31	0.021

Note. *R*^2^ = 0.04.

## Data Availability

Anonymized data files are available via the OSF at https://osf.io/69zjp/?view_only=9e3bdcf3fbed4e9dbce8ca3e370d06f7 (accessed on 27 May 2024).
